# Use of artificial intelligence for liver diseases: A survey from the EASL congress 2024

**DOI:** 10.1016/j.jhepr.2024.101209

**Published:** 2024-09-06

**Authors:** Laura Žigutytė, Thomas Sorz-Nechay, Jan Clusmann, Jakob Nikolas Kather

**Affiliations:** 1 Else Kroener Fresenius Center for Digital Health, Faculty of Medicine and University Hospital Carl Gustav Carus, TUD Dresden University of Technology, Dresden, Germany; 2 Division of Gastroenterology and Hepatology, Department of Internal Medicine III, Medical University of Vienna, Vienna, Austria; 3 Center for Molecular Medicine (CeMM) of the Austrian Academy of Sciences, Vienna, Austria; 4 Christian Doppler Lab for Portal Hypertension and Liver Fibrosis, Medical University of Vienna, Vienna, Austria; 5 Department of Gastroenterology, University Hospital RWTH Aachen, Aachen, Germany; 6 Department of Medicine I, Faculty of Medicine and University Hospital Carl Gustav Carus, TUD Dresden University of Technology, Dresden, Germany; 7 Medical Oncology, National Center for Tumor Diseases (NCT), University Hospital Heidelberg, Heidelberg, Germany

**Keywords:** Deep learning, machine learning, medical data, medical image analysis, large language models, biomarkers, liver cancer, liver fibrosis, liver cirrhosis, MASLD

## Abstract

Artificial intelligence (AI) methods enable humans to analyse large amounts of data, which would otherwise not be feasibly quantifiable. This is especially true for unstructured visual and textual data, which can contain invaluable insights into disease. The hepatology research landscape is complex and has generated large amounts of data to be mined. Many open questions can potentially be addressed with existing data through AI methods. However, the field of AI is sometimes obscured by hype cycles and imprecise terminologies. This can conceal the fact that numerous hepatology research groups already use AI methods in their scientific studies. In this review article, we aim to assess the contemporaneous use of AI methods in hepatology in Europe. To achieve this, we systematically surveyed all scientific contributions presented at the EASL Congress 2024. Out of 1,857 accepted abstracts (1,712 posters and 145 oral presentations), 6 presentations (∼4%) and 69 posters (∼4%) utilised AI methods. Of these, 55 posters were included in this review, while the others were excluded due to missing posters or incomplete methodologies. Finally, we summarise current academic trends in the use of AI methods and outline future directions, providing guidance for scientific stakeholders in the field of hepatology.

**Keypoints**

• Hepatology research has generated vast amounts of complex data suitable for AI analysis, such as images (histopathology and radiology), speech, clinical tabular data, omics, and unstructured medical reports, allowing many research questions to be addressed.
• Studies presented at the EASL Congress 2024 demonstrated the potential of AI in automating and enhancing diagnostics, predicting critical outcomes, stratifying patients, identifying novel biomarkers, and assisting healthcare professionals and patients in navigating the complex landscape of hepatology.
• The implementation of AI in clinical practice faces many challenges, including rigorous prospective validation, regulatory hurdles, and funding issues, which must be addressed to realise the benefits of AI.
• Most of the research presented at the EASL Congress 2024 focused on tabular medical data analysis but with the rise of large language models and advances in the image analysis field through foundation models, growth in their applications can be anticipated.
• Most covered studies focused on metabolic liver disease, fibrosis and cirrhosis, and liver cancer, demonstrating how AI-based methods can extract valuable insights from unstructured visual and textual data.

## Introduction

**Fig. 1:**
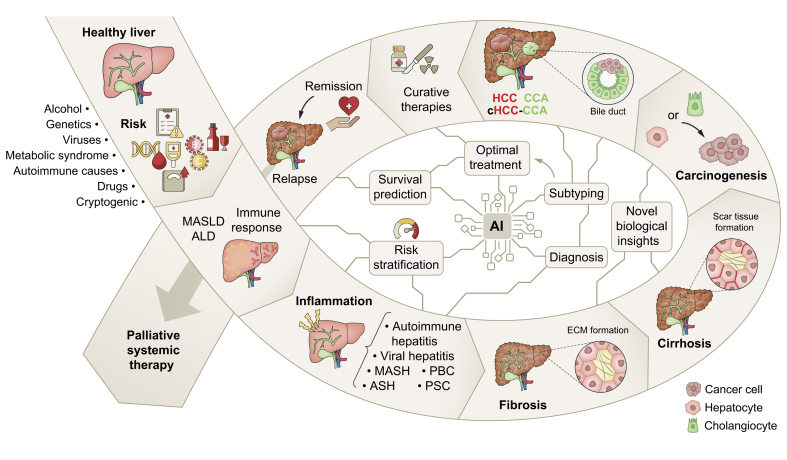
Overview of potential applications of AI in addressing complex and challenging spectrum of liver diseases. Certain risk factors may lead to ALD, MASLD, or immune response, where AI may help predict further progression, thus stratifying patients by risk. Subsequent liver inflammation can progress to hepatic fibrosis and then cirrhosis, conditions marked by an increased risk of liver cancer: HCC, CCA or combined HCC-CCA. AI can assist in differential diagnosis and selecting optimal treatment. AI, artificial intelligence; ALD, alcohol-associated liver disease; ASH, alcohol-associated steatohepatitis; CCA, cholangiocarcinoma; ECM, extracellular matrix; MASH, metabolic dysfunction-associated steatohepatitis; MASLD, metabolic dysfunction-associated steatotic liver disease; PBC, primary biliary cholangitis; PSC, primary sclerosing cholangitis.

**Fig. 2:**
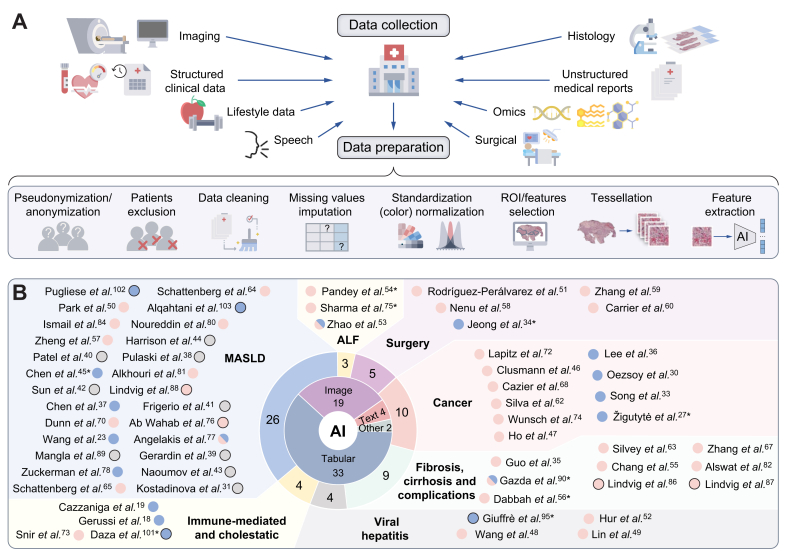
Overview of common clinical data types for AI-based analysis and related studies from the EASL Congress 2024. (A) Many different clinical data types are suitable for AI analysis if appropriately prepared, *e.g*. images might need to undergo ROI selection. (B) Studies included in this review, categorised by application field. The inner donut chart indicates the distribution according to the data modality. Pink dots – machine learning; blue – deep learning; grey – unknown; black border – commercial; ∗ – young investigator. Histopathology slide is from The Cancer Genome Atlas, National Cancer Institute. ALF, acute liver failure; MASLD, metabolic dysfunction-associated steatotic liver disease; ROI, regions of interest.

Chronic liver diseases and cirrhosis cause an increasing number of deaths worldwide^1^ and substantially increase the risk of developing liver cancer, which is the third leading cause of cancer-related death.^2^ Liver diseases remain a major global health concern, especially due to the silent pandemic of metabolic syndrome,^3^ which is a risk factor for various liver diseases. The spectrum of liver diseases is vast and complex, and artificial intelligence (AI) has the potential to be utilised at various stages of diagnosis, treatment, and management (Fig. 1). As AI becomes increasingly integrated into medical research, it could accelerate research efforts and provide new insights across a wide range of medical data modalities, provided the data is correctly prepared for further analysis (Fig. 2A). Many applications of AI technology, specifically in hepatology research, are currently being evaluated.^4^ Furthermore, a recent initiative was launched by the European Association for the Study of the Liver (EASL) governing board – the AI Liver Task Force. Consequently, AI is taking on a more prominent role in hepatology events, including at the largest annual hepatology event in Europe – EASL Congress. We provide an overview of scientific contributions, most of which were presented as posters, and highlights involving AI and its subfields of machine learning (ML) and deep learning (DL) at EASL Congress 2024 in Milan, Italy. Out of 1,857 accepted abstracts (1,712 posters and 145 oral presentations), 6 presentations (∼4%) and 69 posters (∼4%) utilised AI methods. Of these, 55 posters were included in this review, while the others were excluded due to missing posters or incomplete methodologies. All included abstracts and their distribution according to the main research fields are visualised in Fig. 2B.

ML is a subfield of AI that involves training algorithms to make predictions based on mostly tabular data. It encompasses various techniques, such as linear and logistic regression (LR), support vector machine, and variations of decision trees, like random forest classifiers (RFC) and extreme gradient boosting machine (extreme GBM) (Table 1).^5^ Meanwhile, DL is a subset of ML that involves neural networks with many layers and is loosely modelled after the structure and function of the human brain, where neurons work together in large networks to process information.^6^ DL models are designed to automatically learn features and patterns from large amounts of data without explicit human intervention. Depending on the type of data being processed – unstructured text, images, or tabular data – various model architectures can be employed, such as large language models (LLMs),^7^ convolutional neural networks (CNNs),^8^ or vision transformers (Table 2).^9^

**Table 1:** Overview of classical machine learning methods typically applied to tabular data∗ covered in this review.

Method	Description	Advantages	Disadvantages
Decision tree (DT)	Decisions are made by following a path from a starting point to an outcome, based on rules at each step	Interpretable, simple, fast, available feature importance	Cannot capture complex patterns; not effective for imbalanced data; prone to overfit; limited generalization
Random forest (RF)∗∗	An ensemble technique that combines multiple decision trees, introducing randomization to improve accuracy and reduce overfitting	More robust and reliable than single DT; reduced overfitting; computationally inexpensive; available feature importance	Less interpretable than a single DT as one would need to review hundreds of trees; often require manual feature engineering
Gradient boosting machine (GBM)	An ensemble technique that uses multiple decision trees, where each tree corrects the errors of the previous ones based on gradient descent	Tends to achieve higher accuracy than RF; available feature importance scores; versatile due to custom optimisation options	Does not scale well for large datasets; suffer from lower interpretability like RF; prone to overfit; more computationally expensive than RF; sensitive to hyperparameters
Extreme GBM (XGBoost)	Advanced implementation of gradient boosting that is optimised for speed and performance	Increased speed and performance; better control of overfitting; available feature importance scores	Less interpretable than RF; more computationally expensive; requires hyperparameters tuning; prone to overfit if not properly tuned
Support vector machine	Finds the optimal hyperplane to separate different classes in a dataset with the maximum margin	Robust to overfitting if tuned well; versatile and effective for high-dimensional data	Sensitive to outliers; requires tuning; slow with large datasets; less interpretable than simpler methods
Linear regression	Models the relationship between independent input features and a continuous dependent output by fitting a linear equation to the observed data	Simple, efficient, interpretable; available feature importance; less prone to overfit compared to complex models	Cannot capture non-linear relationships; sensitive to outliers; not suitable for highly correlated features
Logistic regression	Models the probability of a categorical outcome using a logistic function (typically binary classification)	Simple; efficient; interpretable; available feature importance; less prone to overfit; can be extended to multiclass classification	Not suitable for capturing complex, non-linear relationships; sensitive to outliers; requires feature engineering; not effective for imbalanced data

∗ The range of suitable data modalities is broader because features can be extracted from images, text, or audio using methods like pretrained models, and then represented in tabular form.

∗∗ RF can also be used for raw images on a pixel level, *e.g*., to classify pixels into foreground/background.

**Table 2:** Overview of deep learning model architectures and machine learning types relevant to this review.

Method	Description	Data modality	Advantages	Disadvantages	Example applications
Shallow artificial neural network (ANN)	A model consisting of interconnected neurons organised in layers, which processes input data to learn patterns and make predictions (typically one or two hidden layers)	Tabular, basic text, simple images	Effective for simple non-linear relationships; can overfit for small datasets	Cannot capture complex patterns; more explainable compared to deep networks	Prediction of clinical targets from other clinical data
Convolutional neural network (CNN)	DL model that uses a sliding window (kernel) to perform convolutional operations and automatically learn pattern detection	Most commonly on images	Automatically learns and extracts features from raw data; effective for images	More computationally expensive than classical ML; require large datasets for training; low interpretability (‘black-box’ models)	Segmentation of images
Transformer	DL model for handling sequential data, like text or image patches, by capturing relationships between all elements in a sequence using self-attention mechanisms	Complex unstructured text data (LLMs); images	Versatile; excel in natural language processing tasks and effective for complex images as well; can scale well to very large datasets	Computationally expensive; require large amounts of data for the training (considerably more than CNNs); complex architectures; very low interpretability (‘black-box’ models)	LLM for extracting structured information from unstructured documents
Supervised learning	The model is trained on labelled data	All	Depends on the AI method used for supervised learning	Require ground truth labels, which can involve manual annotation by an expert	CNN trained on images with annotated regions of interest
Unsupervised learning∗	The model is trained on data without labels	All	No need for labelled data; good for exploratory data analysis and high-dimensional data visualisation	Results heavily depend on data quality; prone to misinterpretation; hard to evaluate	Clustering, dimensionality reduction
Self-supervised learning	The model learns patterns, useful representations or features from unlabelled data, which can then be applied to downstream tasks with labelled data	All	No need for labelled data; trained models can be applied to small datasets for downstream prediction tasks	Very computationally expensive	Histopathology foundation model trained on thousands of unlabelled WSIs
Multiple instance learning (MIL)	The model is trained on "bags" of instances, where each bag is labelled, but the individual instances within the bag are not	All	Useful for medical images where only “weak” labels are available; reduces annotations need	More challenging to interpret results at the bag level; more computationally expensive when datasets are large compared to having all instances labelled	A model is trained to diagnose cancer from a WSI, where not all patches contain tumours

CNN, convolutional neural network; DL, deep learning; LLMs, large language models; ML, machine learning; WSI, whole slide image.

∗ Studies that used only unsupervised learning, *e.g.*, UMAP/t-SNE for data visualisation, were not included in this review.

Here, we aim to provide a straightforward guide to various AI methods and a comprehensive analysis of how these methods are currently shaping hepatology research by exploring the studies presented at the EASL Congress and highlighting the benefits and drawbacks of AI. We have organised the review based on different data modalities (images, tabular data, speech, and text; the number of included studies is displayed in the inner pie chart in Fig. 2B). Within each section, we further categorise the studies according to their objectives, such as AI for predicting outcomes, treatment response, differential diagnosis, risk assessment, biomarker identification, or validation and application of existing AI tools.

## The current landscape of AI in liver diseases

### Clinical image analysis

#### AI for diagnosis

**Fig. 3:**
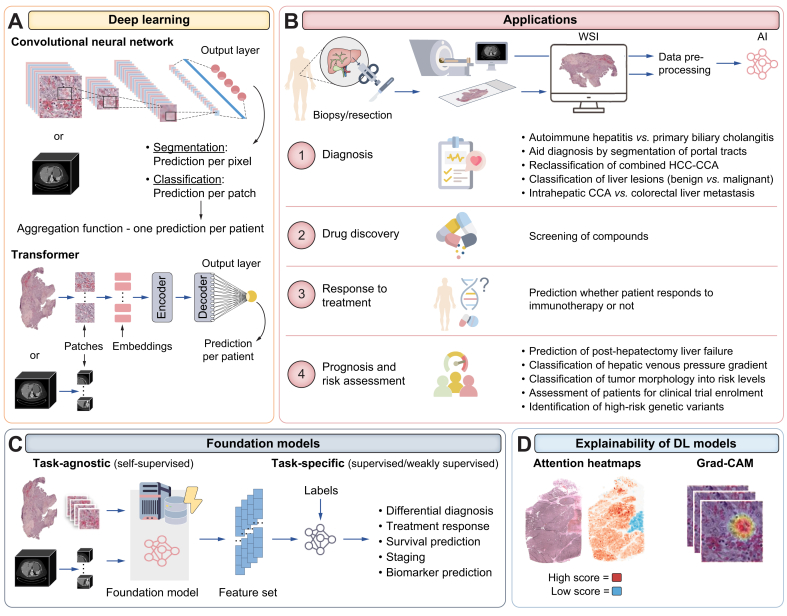
Overview of DL architectures, applications in clinical image analysis, and methods for explainability. (A) The most common architectures of DL models that can be utilised for any image data. (B) Example applications (not all from EASL Congress 2024) of DL models covered in the clinical image analysis section. (C) Foundation models trained in a self-supervised way can be utilised for various downstream prediction tasks in smaller cohorts. (D) Common explainability methods for 'black-box' DL models. The histopathology slide is from The Cancer Genome Atlas. Liver CT is from the open ‘DeepLesion’ dataset.^117^ CCA, cholangiocarcinoma; DL, deep learning; WSI, whole slide image.

Histopathology and radiology image analysis are rapidly evolving fields, driven by great advances in both hardware and sophisticated computer vision techniques. The ability to digitise glass slides of human tissue specimens in high resolution has led to the generation of abundant data that can be effectively mined with AI.^10^ Quality control needs to be assured to avoid issues like out-of-focus areas, sample preparation artifacts, varying colour quality, or compression artifacts.^11^ Additionally, the result may vary depending on the scanner used and the different staining protocols employed at various centres.^12^ However, studies have demonstrated that, provided the quality is adequate, digitised slides do not compromise diagnostic accuracy.^11^ Despite quality assurance and financial challenges in real-world implementation, some pathology laboratories and research groups are increasingly adopting digital workflows to enable AI-based tissue analysis.^13^ Digital histological slides, or whole slide images (WSIs), contain billions of pixels, often posing a challenge due to the huge amounts of information.^14^ Therefore, AI is particularly suitable for analysing such data because it can extract relevant features efficiently. Specifically, DL (Fig. 3A) has been successfully used to accurately discriminate between diseases, types of cancer, or malignant and benign lesions.[15], [16], [17] One notable example from the EASL Congress is a study showing that AI could assist clinicians in differentiating autoimmune liver diseases, such as autoimmune hepatitis (AIH), from primary biliary cholangitis (PBC).^18^ In a multicentre study with an external validation cohort of 92 patients, Gerussi *et al.* demonstrated that PBC could be distinguished from AIH with an AUC of 0.81 in external validation.^18^ The authors used a transformer-based model (Table 2, Fig. 2A) trained on H&E-stained WSIs from 354 patients.^18^ In a smaller cohort of 104 patients, Cazzaniga *et al.* demonstrated that a CNN model (Table 2, Fig. 2A) was able to segment portal tracts in biopsies of autoimmune liver diseases, aiding in diagnosis and reducing inter-observer variability.^19^ In the context of histopathology, these studies show the potential of AI to enhance diagnostic accuracy and enable more standardised and reliable tissue analysis by automating the process and assisting clinicians (Fig. 3B).

Self-supervised learning has become a widely used approach for pretraining foundation models on thousands to tens of thousands of images without annotations or labels, marking an important paradigm shift in the field.^20^^,^^21^ When these foundation models are used to extract features from images for various downstream tasks, such as classification, reduced amounts of labelled data are needed (Fig. 3C). The previously discussed study by Gerussi *et al.* utilised a histopathology foundation model CTransPath^21^ to differentiate PBC from AIH.^18^ Furthermore, not only can this technology be utilised for differential diagnosis, but also to determine the severity of disease. For example, Wang *et al.* utilised a pretrained foundation model^22^ to predict the histological grading of metabolic dysfunction-associated steatohepatitis (MASH) from WSIs with a model trained on 187 and tested on 179 images.^23^ This approach improved the prediction of ballooning and lobular inflammation compared to fully supervised methods, while the accuracy for steatosis remained comparable.^23^ An increasing number of published studies show the potential of foundation models^20^^,^^24^; however, they are currently underrepresented in the field of liver diseases.^4^ Notable examples include the application of foundation models to reclassify combined HCC-cholangiocarcinoma (CCA) with predictions consistent with clinical outcomes.^25^ Other previously published studies typically involve fully supervised methods for diagnostic tasks, such as classifying HCC nodular lesions^16^ or differentiating intrahepatic CCA from colorectal liver metastasis.^26^

The decision-making processes of complex DL models, often called ‘black-box’ models, are not easily interpretable compared to classical ML methods such as decision trees. To address this, our study introduced counterfactual explanations for DL models using generative AI.^27^ By training a diffusion-based autoencoder, we can manipulate patches from WSIs of hepatobiliary cancers to look like the opposite class and observe histopathological features that the model has learned to associate with a particular class (*i.e*., HCC or CCA).^27^ This method can potentially enhance understanding and trust of DL models and ultimately contribute to more transparent AI-driven diagnostics and prognostics in healthcare. Other explainability methods available for DL models are attention heatmaps for transformers or Grad-CAMs for CNNs^28^^,^^29^ (Fig. 3D).

Computed tomography (CT) scans, magnetic resonance imaging (MRI), or contrast-enhanced ultrasound are further imaging modalities that can benefit from AI-based analysis. Advances in AI applications in radiology can potentially enhance diagnostic accuracy, risk assessment, and disease prognosis prediction. For example, Oezsoy *et al.* have shown that AI can assist in classifying benign and malignant liver lesions in routine contrast-enhanced ultrasound data without the need for complex data preprocessing, such as the segmentation of regions of interest, drastically increasing applicability due to the utilisation of the pretrained foundation model.^30^ While not yet externally validated, the model achieved a high AUC of 0.88 on the internal test set.^30^

#### Drug discovery

AI can also be applied in drug discovery for liver diseases (Fig. 3B). For example, Kostadinova *et al.* utilised the proprietary AI imaging platform FibroNest (PharmaNest, US) to study the effects of anti-fibrotic compounds in 3D MASH models using Sirius red staining.^31^ They validated the tool on known anti-fibrotic compounds, showing its potential in drug discovery.^31^ However, few studies address the use of AI in drug discovery for hepatology, which could represent a future area of interest.

#### Prediction of response to treatment

AI also plays a role in predicting responses to specific treatments, for example, in liver cancer (Fig. 3B). Specifically, the prediction of particular gene signatures linked to immunotherapy response from routinely available image data,^32^ *e.g*. in the case of atezolizumab–bevacizumab, has been achieved in the past.^28^ At the EASL congress, one poster presented data on a light GBM model, which was applied to MRI images to predict whether patients would respond to immunotherapy with targeted therapy and chemotherapy (retrospective analysis of a cohort of 116 patients), achieving an AUC of ∼0.86.^33^ This shows potential as a step towards non-invasive personalised medicine; however, extensive external validation is required.

#### Prognosis and risk assessment

Research presented at the EASL congress has shown the promise of using AI on imaging data to predict outcomes and prognosis across various hepatology fields (Fig. 3B). For example, post-hepatectomy liver failure (PHLF) prediction was demonstrated using quantitative features extracted from automatically segmented liver and spleen in gadoxetic acid-enhanced MRI images from 1,760 patients.^34^ Further analysis included a multivariable LR model using clinical and MRI-derived features, which achieved an AUC of 0.78, demonstrating superior performance compared to some other serum biomarker scores and liver function tests.^34^ Additionally, AI models have been utilised to predict hepatic venous pressure gradient from CT scans in 329 patients with cirrhosis, achieving a correlation coefficient of ∼0.50 for the regression task and a high AUC for classifying clinically significant portal hypertension.^35^ Another study demonstrated that DL can classify the tumour morphology of HCC from CT images, typically a time-consuming and labour-intensive task, into different risk levels.^36^ These findings show the potential of AI to improve surgical planning and patient management, ultimately leading to better outcomes.

Furthermore, AI models are addressing the high costs associated with genome analysis by identifying high-risk patients. For example, *PNPLA3* (patatin-like phospholipase domain-containing protein 3) variant carriers are at an increased risk of steatotic liver disease (SLD). Therefore, a UK Biobank data study proposed a DL model that combines a CNN for segmenting liver areas from MRI images and a vision transformer for predicting *PNPLA3* and investigating distinct steatosis distribution patterns.^37^ While the model achieved an AUC of only 0.62 in a test cohort of 2,036 patients with SLD, some distinct patterns could be observed.^37^

Further advances have shown potential in the management of metabolic dysfunction-associated steatotic liver disease (MASLD) and MASH. For example, positive results were reported when using a proprietary AI-based tool (AIM-MASH AI Assist∗, PathAI, US) to assess liver biopsies of 1,451 patients with/without cirrhosis for MASH clinical trial enrolment and endpoint assessment.^38^ In a related study, a proprietary AI-based tool (AIM-MASH, PathAI) and additionally developed ML models (Liver Explore∗, PathAI) were applied to WSIs from 3,577 patients enrolled in MASH clinical trials to characterise fibrosis composition by identifying different cell types and tissue regions^39^. The histological features extracted with these ML models were also correlated with non-invasive tests for monitoring the risk of progression-to-cirrhosis and liver-related events (LREs) in patients with MASH/MASLD and showed better performance than some non-invasive tests.^40^ Furthermore, the AIM-MASH tool’s utility has also been demonstrated in combination with spatial transcriptomics data to show the biological heterogeneity of SLD.^41^ Another proprietary AI-based quantitative assessment tool – qFibrosis (Histoindex, Singapore) – has been utilised to show specific liver lobular changes associated with kidney dysfunction in patients with MASLD.^42^ Naoumov *et al.* investigated how biopsy size affects the reliability of qFibrosis, showing that a minimum size is important for highly reproducible results with reduced sampling errors.^43^ Furthermore, qFibrosis-extracted features were utilised to predict fibrosis progression in the MAESTRO-NASH clinical trial.^44^ Lastly, AI-based image analysis was used to quantify myeloid cell infiltration in H&E-stained liver tissue sections, revealing immunometabolic changes in steatotic livers following dietary intervention and bariatric surgery in rat MASLD models.^45^ These findings highlight the potential of AI-based image analysis to enhance the accuracy and reliability of histologic scoring and characterisation in metabolic liver disease research and clinical trials, as well as to uncover intricate biological changes.

### Tabular data analysis

#### Prognosis and risk stratification

**Fig. 4:**
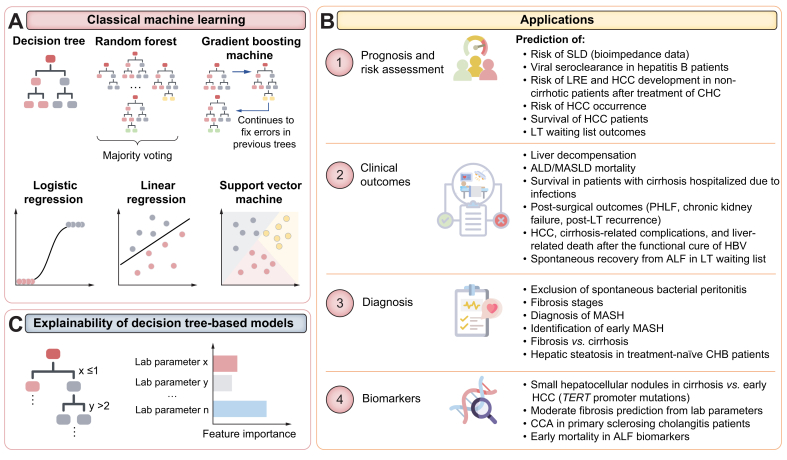
Overview of classical ML methods and their applications. (A) The most popular classical ML methods. (B) Example applications covered in this review utilising these methods (C) Decision tree-based models have greater explainability than deep learning models. ALD, alcohol-associated liver disease; ALF, acute liver failure; CCA, cholangiocarcinoma; CHB, chronic hepatitis B; CHC, chronic hepatitis C; LRE, liver-related events; LT, liver transplantation; MASLD, metabolic dysfunction-associated steatotic liver disease; MASH, metabolic dysfunction-associated steatohepatitis; ML, machine learning; PHLF, post-hepatectomy liver failure; SLD, steatotic liver disease. ∗Indicates a young investigator.

Most AI-based research presented at the EASL Congress was based on applications on tabular data (Fig. 2B), likely due to the abundance of data and lower hardware and computing time requirements. Integrating clinical parameters such as serum data, electronic health records (EHRs), and -omics data enables more accurate predictive models to be built based on patient-specific information. Due to the lower dimensionality of data compared to other modalities like imaging, classical ML methods, such as RFC or LR (Table 1, Fig. 4A), are more frequently employed for tasks where modelling non-linear relationships between variables is needed. The advantage of classical ML methods, like RFC, is their superior interpretability compared to 'black-box' DL models, enabling the identification of the most important features for the prediction (Fig. 4C). For example, Clusmann *et al.* demonstrated that an RFC could achieve an AUC of 0.88 in predicting HCC occurrence.^46^ The authors used data from the UK Biobank containing EHRs, genomics, metabolomics, and lifestyle information from 502,273 patients. Interpretability analyses helped to identify blood and metabolomic parameters as highly relevant for the prediction, followed by lifestyle and EHR parameters, while genomic factors received relatively low importance.^46^ These findings underscore the value of integrating diverse data types and using interpretable ML methods to enhance the performance of risk stratification models. Another study aimed to define a risk score for the survival of patients with HCC (cohort of 4,038 patients).^47^ The authors used LASSO Cox regression to perform feature selection that was subsequently used for the survival analysis, which showed that overall survival could be predicted with an AUC of 0.80 and 5-year survival with an AUC of 0.87–0.89^47^. These studies highlight the potential of ML to predict disease occurrence and provide valuable prognostic information, thereby supporting more personalised and effective patient management strategies in liver cancer (Fig. 4B).

Shifting focus from liver cancer to hepatitis B, Wang *et al.* employed ML techniques to predict viral seroclearance, which shows the potential of AI in helping to understand infection outcomes and aiding in the assessment of novel therapies.^48^ The authors analysed a large, diverse cohort of patients with chronic hepatitis B (CHB) to predict HBsAg seroclearance.^48^ They identified ethnicity, deprivation score, and several routine laboratory parameters as key predictors in addition to already known ones, with the extreme GBM model achieving the highest AUC of ∼0.81^48^. Furthermore, an RFC has been applied to predict the risk of LRE and HCC development in patients without cirrhosis after successful treatment of chronic hepatitis C.^49^

In the context of SLD, Park *et al.* demonstrated a risk prediction LR model using bioimpedance data of over 20,000 adults for the training and over 159,000 for the external validation.^50^ The authors aimed to facilitate early detection and intervention, particularly addressing the challenge of under-screened groups.^50^ The models achieved AUC values of up to 0.85, demonstrating their potential as cost-effective, non-invasive tools for screening.^50^

Accurate risk stratification is essential for prioritising patients awaiting liver transplantation (LT). Rodríguez-Perálvarez *et al.* developed and validated the Gender-Equity Model for Liver Allocation using AI (GEMA-AI), built on a shallow neural network to predict waiting list outcomes more accurately than the traditional linear Cox-regression-based sodium-corrected model.^51^ The GEMA-AI model showed improved discrimination for mortality or delisting due to sickness within 90 days, especially among women and patients with extreme analytical values, demonstrating the potential of AI models in LT prioritisation.^51^

#### Prediction of clinical outcomes

Advances in ML have shown potential in predicting liver-related outcomes in various hepatic conditions (Fig. 4B). For instance, outcomes like HCC, cirrhosis-related complications, and liver-related death after the functional cure of HBV could be predicted with a GBM model trained on variables selected by multivariable Cox analysis of 944 patients, achieving a c-index of 0.84 for the multicentre external validation cohort of 1,102 patients.^52^ In another application, spontaneous recovery from acute liver failure (ALF) in patients listed for LT could be predicted with a neural network classifier, and LR and RFC based on 37 clinical variables.^53^ Furthermore, Pandey *et al.* demonstrated that an ML model predicted ALD mortality with high accuracy and sensitivity using a metabolome panel from 270 patients.^54^ All of these studies underscore the utility of ML models in enhancing the prediction of critical liver-related outcomes, thereby aiding in better patient management and treatment planning.

In the field of MASLD, cirrhosis and related complications, several studies presented at the EASL Congress demonstrated the utility of ML models. For instance, Chang *et al.* reported the prediction of liver decompensation in a multicentre cohort of 872 patients with cirrhosis with an accuracy of ∼89% using RFC on clinical variables and EHR data.^55^ Additionally, Dabbah *et al.* utilised ML algorithms on routine clinical features to predict survival in patients with cirrhosis hospitalised due to infections.^56^ In the context of MASLD, Zheng *et al.* demonstrated mortality prediction in 3,233 patients with MASLD using classical ML tools, with LR achieving the best results (AUC of ∼0.89) and performing better than conventional non-invasive scores.^57^ These approaches underscore the potential of early ML-based assessment in improving the outcomes of patients facing acute complications.

Further advances in ML have also been made in predicting post-surgical outcomes. For example, Nenu *et al.* applied LR to predict PHLF, which showed higher performance (AUC of ∼0.94) compared to non-invasive serum tests and liver stiffness measurement for a cohort of 128 patients with compensated cirrhosis and resected HCC.^58^ Similarly, Zhang *et al.* predicted PHLF using k-means clustering and an LR classifier on a cohort of 197 patients.^59^ The study developed a risk-scoring formula through interpretative analysis of the classifier, achieving an AUC of 0.82.^59^ Additionally, Carrier *et al.* demonstrated the prediction of chronic kidney failure post-LT using ML algorithms such as extreme GBM, generalised linear models, and support vector machine on a cohort of 160 LT recipients.^60^ The extreme GBM model achieved the best performance with an AUC of 0.78, identifying pre-transplant renal failure as the main risk factor.^60^ Moreover, another work utilised the ML-based HepatoPredict^61^ tool, which combines gene expression signatures with clinical variables, to support post-LT stratification of patients with HCC.^62^ This study demonstrated that the algorithm, initially developed for patient selection for LT, could also be used to predict post-LT recurrence using the same biomarkers combined with features obtained from histopathology.^62^ High precision (88%) and recall (90%) were demonstrated in predicting recurrence, showing the potential of ML-based tools to aid in achieving better post-LT outcomes by identifying high-risk patients.^62^ All of these studies indicate that ML models can potentially enhance postoperative management and outcome predictions for patients undergoing curative surgeries.

#### Diagnosis

ML models have also shown promise in enhancing the accuracy and efficiency of diagnosis (Fig. 4B). For example, Silvey *et al.* investigated classical ML models (extreme GBM and LR) for the non-invasive exclusion of spontaneous bacterial peritonitis using 20 readily available clinical parameters in multiple cohorts.^63^ High negative predictive values were shown, suggesting the method’s potential to reliably identify patients with cirrhosis and ascites who can avoid a tap in emergency settings.^63^

In the context of MASLD/MASH, ML models have been used to predict fibrosis stages or to diagnose MASH. For the latter, Schattenberg *et al.* utilised two RFCs trained on a large and a small, more readily available, clinical feature set, to accurately identify early MASH and distinguish fibrosis from cirrhosis.^64^ Both models exhibited strong and comparable performance.^64^ Another study aimed to predict MASH in UK Biobank data.^65^ RFC and CatBoost approaches showed the best classification performance with AUCs over 0.8 and enabled the identification of fat-related features and other predictive variables.^65^

In CHB, the incidence of hepatic steatosis is high,^66^ highlighting the need for cost-effective screening methods. Zhang *et al.* aimed to predict hepatic steatosis in 850 treatment-naïve patients with CHB using a non-invasive ML-based model.^67^ Among nine ML models, the RFC showed superior performance with an AUC of 0.81, higher than the hepatic steatosis index, and led to the identification of serum uric acid as the most important predictive variable.^67^ This study shows the promise of ML-based, cost-effective, and non-invasive tools for managing patients with CHB.

#### Biomarker identification

ML approaches can also be used to analyse various forms of tabular data to identify specific signatures that could be used as biomarkers (Fig. 4B). This can be useful for tasks where differential diagnosis is challenging, *e.g*. between small hepatocellular nodules in cirrhosis and early HCC.^68^ To address this issue, Cazier *et al.* demonstrated the prediction of *TERT* promoter mutations, the most frequent genetic alterations in HCC, in small hepatocellular nodules arising in cirrhosis.^68^ Their analysis identified a peptide signature linked to these mutations, achieving good predictive performance (AUC of 0.73–0.91), and suggested that these biomarkers could be used directly on routine histopathological samples for mutation detection.^68^ These findings underscore the potential of ML in enhancing the accuracy of HCC diagnosis through non-invasive methods.

Biomarkers play an important role in risk stratification of patients with MASLD. The previously published ALADDIN study^69^ introduced an ML-based web calculator to predict moderate fibrosis using routine laboratory parameters with or without vibration-controlled transient elastography (ALADDIN-VCTE). Dunn *et al.* trained classical ML-based ALADDIN models (*i.e.*, RFC, GBM, extreme GBM) on a multicentre cohort of 3,708 patients with MASLD to predict moderate fibrosis.^70^ The ALADDIN-VCTE model achieved an AUC of 0.8 on the external multicentre cohort of 1,289 patients, outperforming FibroScan-AST, while the laboratory parameter ALADDIN model had an AUC of ∼0.76^70^. These ML-based models could facilitate enhanced patient management, particularly following the approval of the first drug for MASH treatment.^71^

The field of autoimmune liver diseases presents another area where classical ML can be utilised. Lapitz *et al.* investigated proteomics of extracellular vesicles in bile to identify diagnostic and predictive biomarkers for CCA in patients with primary sclerosing cholangitis (PSC).^72^ High-throughput data from patients with PSC and PSC-CCA (N = 74) identified 21 diagnostic biomarkers, with ML models demonstrating high accuracy (AUC up to 0.996) in diagnosing and predicting CCA development, surpassing traditional markers.^72^ This liquid biopsy approach may enhance early detection and treatment access for patients with PSC at risk of CCA. Another study demonstrated the application of the elastic net model (*i.e*. a variant of linear regression) to identify serum protein signatures for PSC in a cohort of 75 patients.^73^ Further work by Wunsch *et al.* demonstrated the prediction of PSC-related CCA risk from a combination of clinical and laboratory variables and an autoantibody profile in a cohort of 606 patients.^74^ Multiple classical ML approaches have been investigated, with LR showing the best results, reaching an accuracy of 0.86; hence, it has been implemented as a mobile application.^74^

In ALF, early identification of high-risk patients is important for improving outcomes. Sharma *et al.* used different classical ML methods to accurately predict early mortality in patients with ALF susceptible to infections by integrating lipidomics and mycobiome data.^75^ This enabled the identification of specific lipid species and downregulated proteins that correlate with disease severity and outcomes, thereby improving patient stratification.^75^ Potentially, patients with ALF could be routinely tested for elevated plasma levels of specific lipids and downregulation of the identified protein, allowing for easier identification of high-risk patients needing urgent LT. This approach highlights the utility of integrating ML with biochemical data for improved patient management.

## Validation and application of existing ML-based tools and scores

Some non-invasive ML-based tools are already on the market to help assess patients with liver disease by predicting fibrosis and inflammation from routine biomarkers, such as LiverPRO (Evido, Denmark) and LIVERFASt (Fibronostics, US). These tools were comprehensively evaluated at the EASL Congress for their accuracy, reliability and applicability across different populations and clinical settings. LIVERFASt’s utility was shown for convenient screening of steatosis and early detection of fibrosis in patients with psoriasis.^76^ Another work has demonstrated that a GBM model (CatBoost) trained on FIB-4 score parameters, together with the FIB-4 score itself, reaches a higher AUC of ∼0.79 for the prediction of advanced fibrosis compared to the FIB-4 score (AUC of ∼0.75) on an external cohort of patients with MASLD).^77^ Minor improvements could be achieved by adding synthetic tabular data generated by a generative AI model.^77^ FIB-4 was also compared to a neural network-based approach, where the AI model demonstrated good results (AUC of ∼0.92 compared to 0.78 for FIB-4 score).^78^ Furthermore, FIB-4 was evaluated against a previously published ML-based score^79^ for predicting the severity of MASH and advanced fibrosis, with the ML score showing better performance.^80^^,^^81^ Also, the previously published ML-based FIB-6 score was validated in patients with CHB and MASLD, showing better performance than conventional scores.^82^^,^^83^^,^^84^

Since FIB-4 is recommended as an initial screening tool for clinical trial entry,^85^ one study evaluated FIB-4 against LiverPRO in a cohort of 4,869 patients and showed that LiverPRO reduced the number of false positive cases.^86^ Similarly, Lindvig *et al.* compared FIB-4 and LiverPRO on data from 457,152 patients from the UK Biobank and showed that LiverPRO outperformed FIB-4 in predicting LREs.^87^ The same team has also shown that the MASLD trial recruitment pathway could be optimised by employing LiverPRO.^88^

Other studies are utilising existing AI technologies to extract knowledge from data. For instance, a study identified characteristics related to the risk of long-term liver and cardiovascular outcomes in 13,880 patients with MASH using the AI-based PhenOM platform (OM1 Inc., USA).^89^ The authors identified characteristics strongly associated with liver-related outcomes in patients without cirrhosis, including chronic diseases, kidney issues, and heart issues.^89^ These findings show that AI could assist in identifying patients who would benefit from more intensive monitoring.

## Speech data analysis

The potential benefits of AI extend beyond imaging and tabular data and are becoming evident in other modalities. For instance, Gazda *et al.* demonstrated that classical ML algorithms and DL could detect differences in the speech of patients with hepatic encephalopathy.^90^ However, the study is limited by its small sample size and the recording of specific vowels instead of free or predefined speech.^90^ Nevertheless, it serves as a proof of concept and will be extended to a larger study. The findings of this work indicate a potential future area of interest for this relatively unexplored modality in hepatology.

## Large language models for unstructured data

LLMs have advanced significantly in the past 2 years due to technical improvements that have enhanced their efficiency and effectiveness in language tasks.^91^ These models, for example, GPT4 from OpenAI or Claude-3 from Anthropic, can process input text and generate output that exhibits human-level performance.^92^^,^^93^ Therefore, they can be used for a wide range of tasks in medical research, such as interpretation of medical guidelines,^94^^,^^95^ drug discovery,^96^ developing ML pipelines for clinical studies,^97^ or structuring unstructured information from medical documents.^98^^,^^99^ While previous research has demonstrated the potential of using the retrieval-augmented generation^100^ technique with LLMs to interpret and compare guidelines for HCC, pancreatic, and colorectal cancer,^94^ Giuffrè *et al.* applied retrieval-augmented generation to interpret EASL guidelines on the treatment of hepatitis C.^95^ Another group evaluated four chatbots (ChatGPT 3.5, Claude, Copilot [formerly Bing Chat] from Microsoft, and Gemini [formerly Bard] from Google) focusing on autoimmune liver diseases and the potential of LLMs to assist healthcare professionals.^101^ The authors reported that chatbots showed promising results, with Claude achieving the highest response quality, and highlighted areas needing improvement, such as accuracy and focused, up-to-date information. All of these findings strengthen the evidence of LLMs’ potential to assist healthcare professionals in navigating the increasingly complex landscape of liver diseases.

Furthermore, evidence shows that LLMs could be helpful for patients by providing information about liver diseases, offering detailed explanations, answering questions, and assisting in understanding complex medical information. Two studies at the EASL Congress evaluated the capability of ChatGPT 3.5 as a counselling tool for Italian-speaking and Arabic-speaking patients with MASLD.^102^^,^^103^ Both groups found the model’s answers complete and comprehensive; however, the Italian group reported suboptimal accuracy in certain domains. Nevertheless, with further validation studies, LLMs have the potential to become a valuable support tool for patients suffering from liver diseases.

Although LLMs exhibit great potential, their outputs should be interpreted cautiously and not considered medical advice at this time.^104^^,^^105^ Since the best-performing LLMs are proprietary, it is essential to avoid sharing sensitive or personal information. Only open-source LLMs, such as Llama and Falcon models, can be hosted on a private server and used without privacy concerns, though this requires powerful hardware. The performance of these models can be continuously improved with specific prompt engineering techniques^106^ such as emotional stimuli,^107^ chain-of-thought,^108^ thread-of-thought,^109^ or chain-of-verification.^110^ Furthermore, LLMs can summarise vast amounts of information, aiding in writing literature reviews, screening and selecting papers, and automating data extraction.^111^ However, there are many pitfalls of using AI for such tasks, including the lack of sources for the model’s outputs, “hallucinations” (*i.e.* producing factually incorrect statements that appear plausible), and answers based on outdated information.^112^ These pitfalls could be mitigated by creating guidelines for using generative AI in research and educating researchers in AI literacy, including the responsible use of these technologies.

## Challenges, limitations and gaps

**Fig. 5:**
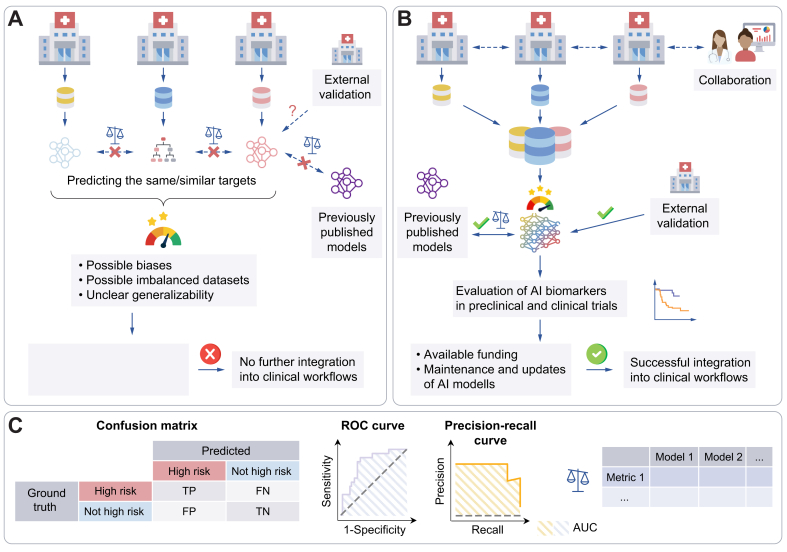
Limitations of many studies incorporating AI and how to overcome them. (A) Many studies use rather small cohorts, lack external validation, and do not compare methods with published approaches, hindering clinical implementation. (B) Collaborative efforts are needed to build multicentre cohorts to develop reliable AI models that could be further validated in (pre)clinical trials. (C) Selecting the right evaluation metrics is important; for example, the precision-recall curve is better for imbalanced data as it highlights positive class performance and is not influenced by many TN. FN, false negatives; FP, false positives; TN, true negatives; TP, true positives.

Despite the great promise of AI in liver research, several limitations have delayed its widespread adoption in clinical practice (Fig. 5A). One issue is the small sample sizes often used in studies and the lack of external validation cohorts. This can lead to models that perform well on internal validation but fail when applied to independent external cohorts. The lack of robust testing raises concerns about the generalisability and reliability of these models in diverse clinical settings. However, acquisition of large datasets needed to train AI models is challenging. A major challenge stems from different data protection, privacy, and research ethics laws across different jurisdictions.^113^ There is a notable lack of multicentric and multinational studies involving AI due to these data-sharing challenges. One of the possible solutions to overcome this barrier to data accessibility in research is to utilise federated or swarm learning.^114^ These approaches allow models to be trained across multiple centres without the need to share sensitive patient data, thereby respecting privacy and regulatory constraints. Alternatively, a more straightforward approach when multicentric studies are not possible could be to open source the weights of trained AI models. This would allow subsequent independent studies to compare models, validate their performance, and investigate potential issues such as biases.

Collaborative efforts between researchers and clinicians with similar aims should be encouraged in order to facilitate the development of reliable and universally applicable diagnostic and prognostic AI tools (Fig. 5B). Furthermore, careful consideration should be given when selecting evaluation metrics, as the precision-recall curve is more informative for imbalanced datasets than the most commonly used receiver-operating characteristic curve (true positive rate *vs*. false positive rate) (Fig. 5C).^115^ Such datasets are common in medical research, where rare events are frequently outnumbered by instances from the healthy population. The precision-recall curve evaluates the fraction of true positives among all positive predictions and is, therefore, more reliable for assessing classifier’s performance when predicting rare classes.

Another obstacle is that implementing AI in clinical practice involves substantial initial and ongoing expenses, presenting challenges for funding despite promising long-term cost efficiencies and better patient outcomes.^13^ Careful consideration of reimbursement and other strategies are needed to mitigate this and support widespread adoption. Finally, regulatory frameworks must evolve to keep pace with the rapid development of AI technologies, ensuring effective and ethical integration into healthcare.^116^

A particular domain that has not yet reached its full potential is the use of LLMs for liver health, where these models could be leveraged to analyse large quantities of unstructured text data, such as clinical reports and medical guidelines. LLMs could be used to extract relevant information from multiple medical guidelines that often contain varying recommendations.^94^ Patient engagement is another area where the potential of LLMs remains under-explored. Further research could explore how these technologies can enhance communication, education, and shared decision-making between patients and clinicians.

## Engaging the community

The adoption of AI technologies in liver healthcare is dependent on engagement across all levels – from patients to clinicians making the decisions and scientists. Therefore, it is valuable to communicate the benefits and limitations of these technologies, for example, during talks at congresses and conferences, in reviews of published research, or through other formats that are more accessible to patients. Further patient engagement could potentially be achieved through collaboration with patient associations like the European Liver Patients' Association (or ELPA).

## Conclusions

The use of AI in the management of liver diseases is an advancing area of research owing to numerous clinical needs and the complexity of clinical decision-making. Research presented at the EASL Congress shows the potential of AI in automating diagnostics, predicting critical outcomes, stratifying patients, identifying novel biomarkers, and assisting healthcare professionals and patients in navigating the complex landscape of hepatology (highlighted studies in Table 3). By leveraging various data types, AI-based models can improve patient management and treatment planning in various hepatic conditions by making more accurate prognostic assessments that could lead to better targeted interventions. Our review shows that MASLD remains a hot topic in the liver AI field, closely followed by cancer research and liver fibrosis (Fig. 2B). ML methods focused on tabular data were the most commonly utilised AI tools, followed by DL on image data. Recent advances in LLMs have driven the increased use of text data, a trend that is expected to continue. However, certain issues need to be addressed before successful implementation in clinical practice can occur. They can be overcome by collaborative efforts, robust validation of developed models in multicentre and prospective studies, and regulatory adaptation.

**Table 3:** Summary of selected key studies presented at the EASL Congress 2024 applying AI in liver disease research.

Study	Modality	Approach	Patient number	Application
Gerussi *et al.*^18^	Histopathology	DL: Transformer+MIL	Training/int. val. 354; ext. val. 92	Differentiate AIH from PBC
Gazda *et al.*^90^∗	Speech data	Classical ML: extreme GBM & DL: EfficientNet	62	Diagnose hepatic encephalopathy
Rodríguez-Perálvarez *et al.*^51^	Clinical variables	DL: ANN	Training/int. val. 5,761/1,920; ext. val. 1,638	Gender-equity model for liver allocation for liver transplant candidates
Silvey *et al.*^63^	Clinical variables	Classical ML: extreme GBM, LR	Training/int. val. 7,233/2,410; ext. val. 2,844 & 333	Differentiate patients with SBP
Zhao *et al.*^53^∗	Clinical variables	Classical ML: RF, LR & DL: ANN	2,347	Identify spontaneous recovery in ALF
Wang *et al.*^48^	Clinical variables	Classical ML: extreme GBM, RF, LR, GBDT	4,471	Prediction of hepatitis B surface antigen seroclearance

AIH, autoimmune hepatitis; ALF, acute liver failure; ANN, artificial neural network; DL, deep learning; ext. val., external validation; GBDT, gradient boosted decision tree; GBM, gradient boosting machine; int. val, internal validation; LR, logistic regression; MIL, multiple instance learning; ML, machine learning; PBC, primary biliary cholangitis; RF, random forest; SBP, spontaneous bacterial peritonitis. ∗Indicates a young investigator.

## Abbreviations

ALF, acute liver failure; ALD, alcohol-associated liver disease; AI, artificial intelligence; AIH, autoimmune hepatitis; CCA, cholangiocarcinoma; CHB, chronic hepatitis B; CNN, convolutional neural network; CT, computed tomography; DL, deep learning; EHRs, electronic health records; EASL, European Association for the Study of the Liver; GBM, gradient boosting machine; LLM, large language model; LREs, liver-related events; LT, liver transplantation; LR, logistic regression; ML, machine learning; MRI, magnetic resonance imaging; MASH, metabolic dysfunction-associated steatohepatitis; MASLD, metabolic dysfunction-associated steatotic liver disease; PHLF, post-hepatectomy liver failure; PBC, primary biliary cholangitis; PSC, primary sclerosing cholangitis; RFC, random forest classifier; SLD, steatotic liver disease; VCTE, vibration-controlled transient elastography; WSI, whole slide image.

## Financial support

JC is supported by the Mildred-Scheel-Postdoktorandenprogramm of the German Cancer Aid (grant 70115730). JNK is supported by the German Cancer Aid (DECADE, 70115166), the German Federal Ministry of Education and Research (PEARL, 01KD2104C; CAMINO, 01EO2101; SWAG, 01KD2215A; TRANSFORM LIVER, 031L0312A; TANGERINE, 01KT2302 through ERA-NET Transcan; Come2Data, 16DKZ2044A; DEEP-HCC, 031L0315A), the German Academic Exchange Service (SECAI, 57616814), the German Federal Joint Committee (TransplantKI, 01VSF21048) the European Union’s Horizon Europe and innovation programme (ODELIA, 101057091; GENIAL, 101096312), the European Research Council (ERC; NADIR, 101114631), the National Institutes of Health (EPICO, R01 CA263318) and the National Institute for Health and Care Research (NIHR, NIHR203331) Leeds Biomedical Research Centre. The views expressed are those of the author(s) and not necessarily those of the NHS, the NIHR or the Department of Health and Social Care. This work was funded by the European Union. Views and opinions expressed are those of the author(s) only and do not necessarily reflect those of the European Union. Neither the European Union nor the granting authority can be held responsible for them.

## Conflict of interest

JNK declares consulting services for Bioptimus, France; Owkin, France; DoMore Diagnostics, Norway; Panakeia, UK; AstraZeneca, UK; Mindpeak, Germany; and MultiplexDx, Slovakia. Furthermore, he holds shares in StratifAI GmbH, Germany, Synagen GmbH, Germany, and has received a research grant by GSK, and has received honoraria by AstraZeneca, Bayer, Daiichi Sankyo, Eisai, Janssen, Merck, MSD, BMS, Roche, Pfizer and Fresenius.

Please refer to the accompanying ICMJE disclosure forms for further details.

## Authors’ contributions

Laura Žigutytė: Conceptualization, Writing - Original Draft, Writing – review & editing, Visualisation. Thomas Sorz-Nechay: Conceptualization, Writing – review & editing, Visualisation. Jan Clusmann: Conceptualization, Writing – review & editing. Jakob Nikolas Kather: Conceptualization, Supervision, Funding acquisition, Writing – review & editing.

## Declaration of Generative AI and AI-assisted technologies in the writing process

During the preparation of this work the author(s) used Grammarly and ChatGPT 4o in order to correct the grammar, clarity, and conciseness of the text. After using this tool/service, the author(s) reviewed and edited the content as needed and take(s) full responsibility for the content of the published article.
