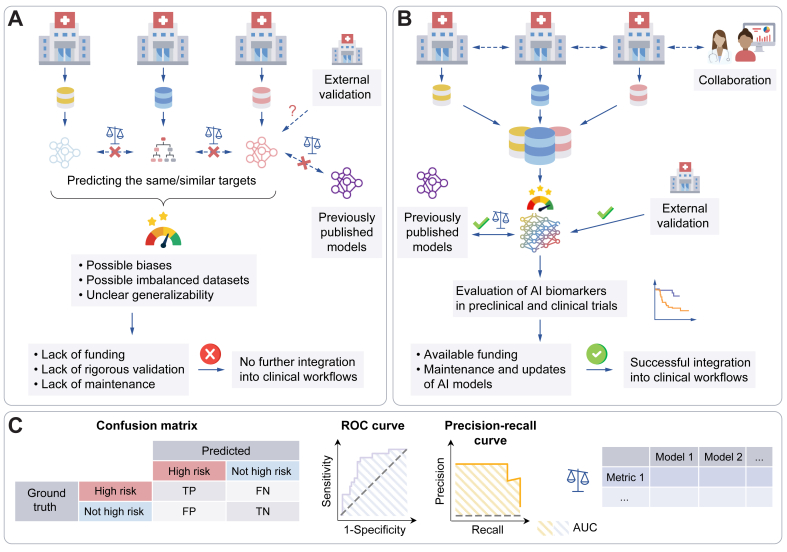# Erratum to ‘Use of artificial intelligence for liver diseases: A survey from the EASL congress 2024’ [JHEP Reports (2024) doi https://doi.org/10.1016/j.jhepr.2024.101209]

**DOI:** 10.1016/j.jhepr.2025.101446

**Published:** 2025-05-22

**Authors:** Laura Žigutytė, Thomas Sorz-Nechay, Jan Clusmann, Jakob Nikolas Kather

**Affiliations:** 1 Else Kroener Fresenius Center for Digital Health, Faculty of Medicine and University Hospital Carl Gustav Carus, TUD Dresden University of Technology, Dresden, Germany; 2 Division of Gastroenterology and Hepatology, Department of Internal Medicine III, Medical University of Vienna, Vienna, Austria; 3 Center for Molecular Medicine (CeMM) of the Austrian Academy of Sciences, Vienna, Austria; 4 Christian Doppler Lab for Portal Hypertension and Liver Fibrosis, Medical University of Vienna, Vienna, Austria; 5 Department of Gastroenterology, University Hospital RWTH Aachen, Aachen, Germany; 6 Department of Medicine I, Faculty of Medicine and University Hospital Carl Gustav Carus, TUD Dresden University of Technology, Dresden, Germany; 7 Medical Oncology, National Center for Tumor Diseases (NCT), University Hospital Heidelberg, Heidelberg, Germany

It has come to our attention that a formatting error and typo were introduced into Fig. 5 of our manuscript during the production process. In Fig. 5A, the text was missing from the box at the bottom of the panel. In Fig. 5B, ‘models’ was misspelled ‘modells’. These errors have been corrected in the online version. The publisher apologises for any inconvenience caused.

**Figure:**